# Bilateral vestibulopathy in *RFC1*-positive CANVAS is distinctly different compared to *FGF14*-linked spinocerebellar ataxia 27B

**DOI:** 10.1007/s00415-023-12050-0

**Published:** 2023-10-20

**Authors:** Max Borsche, Mirja Thomsen, David J. Szmulewicz, Bente Lübbers, Frauke Hinrichs, Paul J. Lockhart, Katja Lohmann, Christoph Helmchen, Norbert Brüggemann

**Affiliations:** 1 Institute of Neurogenetics, University of Lübeck and University Hospital Schleswig-Holstein, Campus Lübeck, Lübeck, Germany; 2 Department of Neurology, University of Lübeck and University Hospital Schleswig-Holstein, Campus Lübeck, Ratzeburger Allee 160, 23538 Lübeck, Germany; 3 Cerebellar Ataxia Clinic, Eye and Ear Hospital, Melbourne, VIC Australia; 4 The Bionics Institute, University of Melbourne, Melbourne, VIC Australia; 5 Bruce Lefroy Centre, Murdoch Children’s Research Institute, Parkville, VIC Australia; 6 Department of Paediatrics, University of Melbourne, Parkville, VIC Australia

Dear Sirs,

The recent discoveries of two new repeat expansion disorders for late-onset cerebellar ataxia (CA), frequently accompanied by vestibulopathy and neuropathy, shed new light on ataxia research. Biallelic intronic repeat expansions in the *RFC1* (replication factor C subunit) gene [1, 2] are disease causing in about 90% of Cerebellar ataxia, Neuropathy, and Vestibular Areflexia Syndrome (CANVAS) patients [3, 4], while heterozygous intronic repeat expansions in the Fibroblast Growth Factor 14 (*FGF14*) gene explain a relevant number of late-onset CA cases [5–7]. Bilateral vestibulopathy (BV) is a characteristic feature of the CANVAS phenotype of *RFC1-*linked disease, and emerging data suggest that *FGF14* repeat expansion carriers may also exhibit vestibular hypofunction [5, 6, 8]. However, differences in the severity of BV, potentially allowing conclusions about the underlying etiology, are yet to be compared between *FGF14* and *RFC1* repeat expansion carriers. Here, we investigated the role of BV in the differential diagnosis between *RFC1-*, *FGF14-*expansion*-*positive, and repeat-expansion-negative CA patients.

Patients were recruited through the outpatient clinics at the tertiary referral centers for ataxia and vertigo at the University of Lübeck, Lübeck, Germany. The inclusion criterion was the presence of CA (clinically defined by limb ataxia and/or the presence of cerebellar oculomotor signs), whereas patients with secondary forms (toxic, inflammatory, and paraneoplastic) and known repeat-expansion spinocerebellar ataxias (SCA1, 2, 3, 6, and 17) were excluded. The video head-impulse test (vHIT) [9] was carried out in all patients, and caloric response testing [10] was available in a subset. Genetic and some phenotypic data of several of our *RFC1 *(19/25, 76%) [4, 9] and of all *FGF14* [5] repeat expansion carriers have been published before, but here we expand the phenotypic data.

All patients underwent genetic testing for *RFC1* and *FGF14* repeat expansions. For *RFC1*, we applied genetic analyses as described [4], including duplex PCR and Sanger sequencing. For *FGF14*, we performed long-range PCR followed by fragment length analysis as well as repeat-primed PCR and Sanger sequencing. *FGF14* repeat expansions were considered disease related with a repeat number > 250 [5]. Of note, patients with interrupted GAA repeat expansions/non-GAA repeat expansions were excluded [11, 12].

The sample was subdivided by genetic results in *RFC1-*expansion-positive, *FGF14-*expansion*-*positive, and *RFC1-* and *FGF14-*expansion-negative individuals. Bilateral vestibulopathy was defined by a mean vestibulo-ocular reflex (VOR) gain < 0.7 assessed by horizontal video head impulse testing [9]. In brief, eye and head movements were recorded by a digital video camera (Eye-SeeCam HIT System, Autronics, Hamburg, Germany) at a sampling rate of 220 Hz. At least ten passive and rapid (peak velocity 250°/sec) head movements of small amplitude (10–15°) were performed per side. Head impulses were unpredictable in direction and amplitude. The gain of the horizontal vestibulo-ocular reflex was analyzed at a narrow time interval of 60 ± 10 ms after head movement onset. Only the horizontal vestibulo-ocular reflex was analyzed. Somatosensory impairment (SS) was defined by nerve conduction studies, available for 47/58 patients (81%) or clinically by the presence of a reduced vibrational sense and impaired sense of position at the metatarsophalangeal joint. The presence of BV and SS allowed the clinical differentiation in CANVAS (CA + BV + SS), Cerebellar Ataxia with Bilateral Vestibulopathy (CABV; CA + BV), Cerebellar Ataxia with Somatosensory impairment (CASS; CA + SS), and isolated CA.

Mean ± standard deviation (SD) and the frequency of individuals with percentages are shown. Differences between the groups were analyzed dependent on the data distribution by ANOVA or Kruskal Wallis tests, and, if significant and relevant, post hoc tests were applied. We analyzed the utility of the VOR gain to discriminate between *RFC1*-expansion-positive and *RFC1*-expansion*-*negative CABV patients using a receiver operating characteristic (ROC) curve. *p* values < 0.05 were considered significant. Analyses were performed using GraphPad Prism 9 and *jamovi* Version 2.3.

Of the 58 patients (17 females, 29%) investigated within this study, 25 were *RFC1*-expansion positive, nine *FGF14*-expansion positive, and 24 *RFC1* and *FGF14*-repeat expansion negative. Age at examination, age at onset, and disease duration of the whole sample were 72.3 ± 9.6 years, 64.6 ± 10.3 years, and 8.6 ± 6.3 years, respectively. *RFC1*-expansion-positive individuals (67.4 ± 7.9 years) were younger at examination than *FGF14*-expansion-positive (70.7 ± 11.7 years) and repeat-expansion-negative participants (77.9 ± 7.6 years) (Kruskal–Wallis test: *p* < 0.001). Likewise, disease onset was earlier in *RFC1*- (59.3 ± 8.1 years) compared to *FGF14*-expansion-positive patients (62.3 ± 10.7 years) and repeat-expansion-negative individuals (70.9 ± 8.9 years) (Kruskal–Wallis test: *p* < 0.001), leading to a similar disease duration (Kruskal–Wallis test: *p* = 0.06) among all study groups (Table 1).

**Table 1:** Demographics of the study sample, clinical, and genetic findings

	*RFC1-*expansion positive	*FGF14-*expansion positive	Repeat-expansion negative
*N*	25	9	24
Females	18 (72%)	7 (78%)	16 (67%)
AAE in years (mean ± SD)	67.4 ± 7.9	70.7 ± 11.7	77.9 ± 7.6
AAO in years (mean ± SD)	59.3 ± 8.1	62.3 ± 10.7	70.9 ± 8.9
DD in years (mean ± SD)	9.7 ± 4.7	9.1 ± 10.1	7.3 ± 6.1
Cerebellar oculomotor signs	24 (96%)	9 (100%)	23 (96%)
DBN	13 (52%)	9 (100%)	12 (50%)
Dysarthria	14 (56%)	1 (11%)	8 (33%)
Clinical picture			
CANVAS	25 (100%)	2 (22%)	5 (21%)
CABV	0	4 (44%)	8 (33%)
CASS	0	0	4 (17%)
CA	0	3 (33%)	7 (29%)
Mean VOR gain (all; mean ± SD)	0.19 ± 0.12	0.65 ± 0.22	0.70 ± 0.18
Individuals with BV	25 (100%)	6 (67%)	13 (54%)
Mean VOR gain (BV; mean ± SD)	0.19 ± 0.12 (*n* = 25)	0.52 ± 0.14 (*n* = 6)	0.58 ± 0.14 (*n* = 13)

Cerebellar oculomotor signs were defined as the presence of saccadic pursuit, gaze-evoked horizontal or downbeat nystagmus

Number of individuals and frequencies in percent are displayed. Mean VOR is shown for all individuals as well as for individuals with confirmed BV (mean VOR gain of < 0.7) only. For AAE, AAO, and DD, mean ± standard deviation is shown

*RFC1* gene encoding replication factor C subunit 1, *FGF14* gene encoding Fibroblast Growth Factor 14, *AAE* age at examination, *SD* standard deviation, *AAO* age at onset, *DD* disease duration, *DBN* downbeat nystagmus, *CANVAS* cerebellar Ataxia, neuropathy, and vestibular areflexia syndrome, *CABV* cerebellar ataxia with bilateral vestibulopathy, *CASS* cerebellar ataxia with somatosensory impairment, *CA* isolated cerebellar ataxia, *VOR* vestibulo-ocular reflex, *BV* bilateral vestibulopathy

All *RFC1*-expansion-positive patients clinically exhibited the CANVAS phenotype (25/25, 100%). Two *FGF14* repeat expansion carriers (*n* = 9) had a CANVAS phenotype (22%), four had CABV (44%), and three had isolated CA (33%). Repeat-expansion-negative individuals (*n* = 24) clinically presented with the following phenotypes: CANVAS (*n* = 5, 21%), CABV (*n* = 8, 33%), CASS (*n* = 4, 17%), or isolated CA (*n* = 7, 29%).

Cerebellar oculomotor signs (downbeat nystagmus, gaze-holding deficits, and impaired smooth pursuit) were likewise frequent in *RFC1* (96%) and *FGF14* (100%) repeat expansion carriers. We observed dysarthria in 56% of *RFC1-* and 11% of *FGF14-*expansion-positive individuals (Table 1). Albeit not investigated systematically, 14/15 (93%) *RFC1*-expansion-positive individuals reported chronic cough, whereas this feature was not reported by any of the *FGF14* repeat expansion carriers. Hearing ability was not investigated with quantitative measures, but none of the participants had clinically obvious severe hearing loss.

BV, investigated by vHIT, was found in 25/25 (100%) *RFC1*-expansion-positive patients, 6/9 (67%) *FGF14*-expansion-positive patients, and 13/24 (54%) repeat-expansion-negative CA patients. Caloric irrigation testing in individuals with reduced angular VOR gain upon vHIT was available in 5/6 (83%) *FGF14* and 12/25 (48%) *RFC1* repeat expansion carriers with BV. Two of five (40%) *FGF14* and 12/12 (100%) *RFC1* repeat expansion carriers with BV showed bilateral caloric hyporesponsiveness (< 5°/sec). While age at examination differed between *RFC1*-expansion-positive (67.4 ± 7.9 years), *FGF14*-expansion-positive (69.3 ± 10.9 years), and repeat-expansion-negative individuals (78.8 ± 7.3 years) (ANOVA: *p* < 0.01), a correction was not required as VOR gain (as measured by vHIT) does not decrease with age [13]. Disease duration, already demonstrated to influence BV in *RFC1* repeat expansion carriers [9], was similar between the three groups (*RFC1*: 9.7 ± 4.7 years; *FGF14*: 8.0 ± 10.0 years; repeat-expansion-negative: 8.6 ± 7.6 years) (Kruskal–Wallis test: *p* = 0.13). However, VOR gain in *RFC1*-expansion*-*positive individuals was lower as compared to *FGF14*-expansion-positive and repeat-expansion-negative patients (*RFC1*: 0.19 ± 0.13; *FGF14*: 0.52 ± 0.14; repeat-expansion negative: 0.58 ± 0.14) with BV (ANOVA: *p* < 0.0001, for post hoc tests see Fig. 1A). Investigating the capacity of the VOR gain to distinguish *RFC1-*expansion-positive from *FGF14*-expansion-positive and repeat-expansion-negative cerebellar ataxia patients with BV, we found a high accuracy (Area under the Receiver Operator Curve (ROC): 0.97, *p* < 0.0001) in predicting *RFC1*-expansion positivity (Fig. 1B). Illustrating vHIT results of one *RFC1-*expansion-positive individual and one *FGF14* expansion carrier are shown in Fig. 2.

**Fig. 1:**
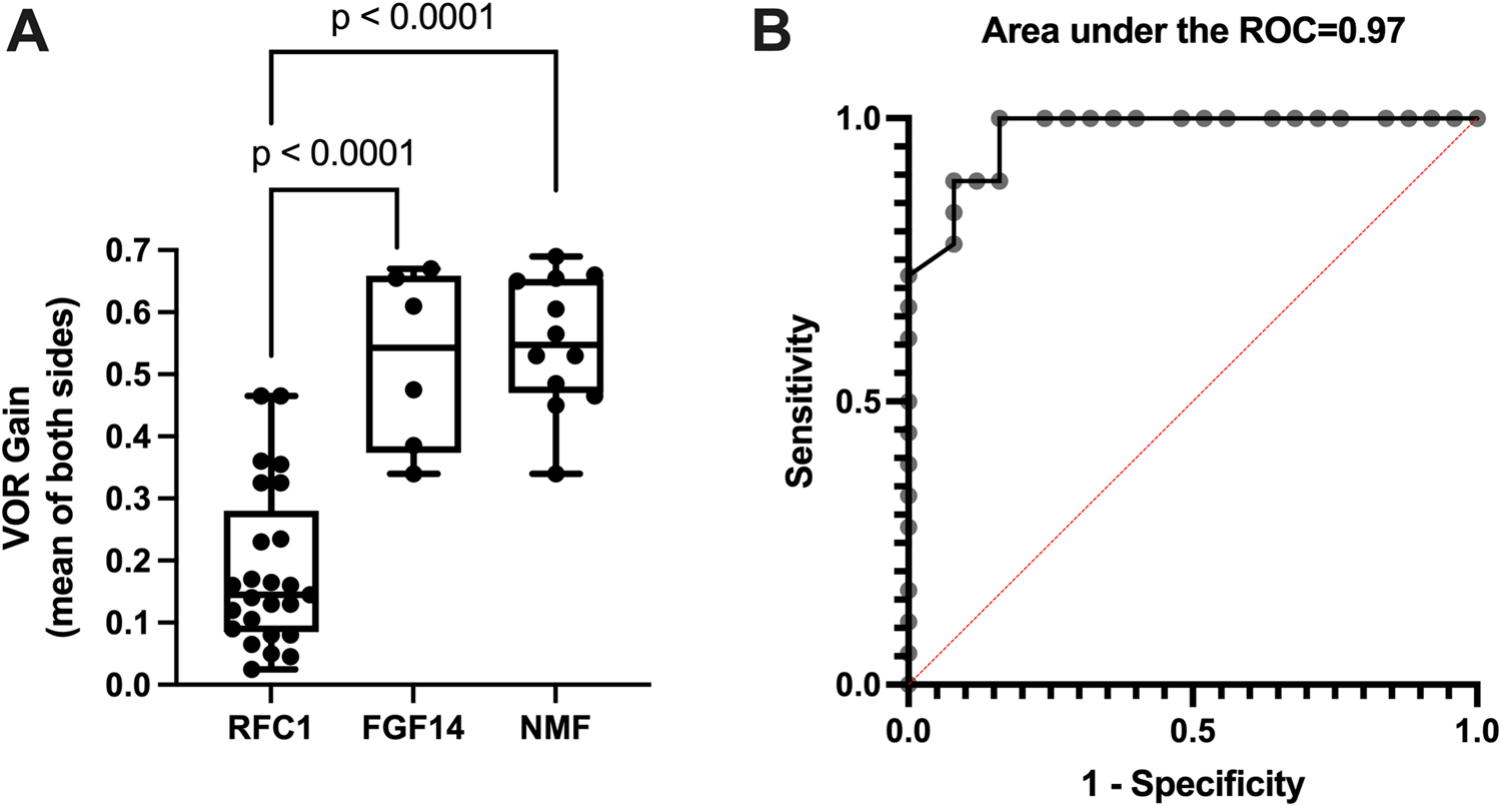
BV (box plot of the mean horizontal vestibulo-ocular reflex (VOR) gain of both sides, assessed by video-head-impulse test) in *RFC1-*expansion-positive, *FGF14*-expansion-positive, and repeat-expansion-negative late-onset cerebellar ataxia patients. Only individuals with a confirmed mean VOR gain of < 0.7 were included in the analysis. **A** VOR gain is lower in *RFC1* repeat expansion carriers (*n* = 25) compared to *FGF14*-positive patients (*n* = 6) and repeat-expansion-negative cerebellar ataxia patients (*n* = 12). *p* < 0.001 (ANOVA), *p* values of post-hoc tests are shown in the figure. **B** Assessment of the utility of the VOR gain to discriminate between *RFC1* repeat expansion carriers (*n* = 25) and *FGF14* repeat expansion carriers/repeat-expansion-negative cerebellar ataxia patients with confirmed BV (*n* = 18) investigated by a receiver operator characteristic (ROC) curve. *p* < 0.0001. *RFC1* gene encoding replication factor C subunit 1, *FGF14* gene encoding Fibroblast Growth Factor 14, *NMF* patients with cerebellar ataxia and BV negative for *RFC1* and *FGF14* repeat expansions, *ROC* receiver operator curve. The figure was created with GraphPad Prism 9

**Fig. 2:**
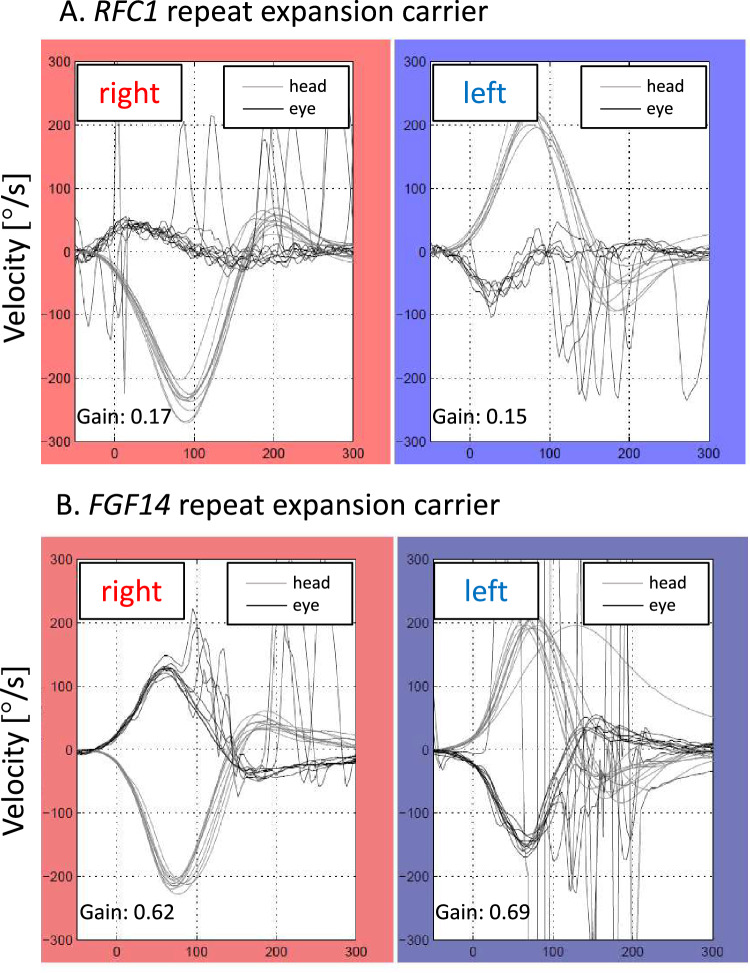
Illustrating video head-impulse test (vHIT) results of one *RFC1* and one *FGF14* repeat expansion carrier with similar age at onset and age at examination. **A** vHIT examination of a 66-year-old *RFC1*-repeat expansion-positive female with five years disease duration until vHIT was performed (age at onset 61 years). At the time of examination, she had downbeat nystagmus, saccadic pursuit, severe dysarthria, as well as limb, stance, and gait ataxia. Nerve conduction studies confirmed sensory neuropathy corresponding to a CANVAS (Cerebellar Ataxia, Neuropathy, and Vestibular Areflexia Syndrome) phenotype. In keeping with the diagnosis of *RFC1*-linked disease, she had a chronic cough. A mean VOR gain of 0.16 at 60 ms showed severe VOR impairment. **B** vHIT examination of a 68-year-old male *FGF14* repeat expansion carrier with likewise five years disease duration until the vHIT was performed (age at onset 63 years). During examination, he had mild dysarthria, gait disturbance, limb and gait ataxia, gaze-evoked horizontal nystagmus, and impaired VOR suppression. Neuropathy was excluded by nerve conduction studies, leading to a CABV (Cerebellar Ataxia with Bilateral Vestibulopathy) phenotype. A mean VOR gain of 0.66 at 60 ms revealed mild VOR impairment upon vHIT testing

Our study revealed a mildly reduced angular VOR gain in two-thirds of *FGF14* repeat expansion carriers, which is in line with the two initial studies, reporting BV in a subset of *FGF14* repeat expansion carriers, and a recent study on an independent cohort, where 75% of *FGF14*-expansion-positive patients had BV [8]. Furthermore, BV was present in all *RFC1* repeat expansion carriers where the severity of BV was significantly greater compared to *FGF14*-expansion-positive individuals as well as in repeat-expansion-negative CA patients. This finding is important for at least two reasons: First, our data suggest that the severity of BV aids in clinically distinguishing *RFC1*-expansion-positive from *RFC1-*expansion*-*negative ataxic individuals (including *FGF14* repeat expansion carriers) with high accuracy. Second, this difference in the magnitude of VOR gain reduction intimates a potentially different pathophysiological underpinning for the VOR gain reduction in *RFC1*- and *FGF14*-linked disease. One option is that the markedly reduced angular VOR gain (< 0.5) reflects peripheral vestibular organ (neuropathy/ganglionopathy) lesions and is hence associated with caloric hyporesponsiveness. In contrast, a mildly pathological vHIT result may indicate cerebellar (floccular) pathology which may not be present on caloric testing [14]. We do however note that experimental floccular damage may increase, decrease, or have no effect on VOR gain [15]. Degeneration of the vestibular ganglia is well established for CANVAS [16]. Although histological analyses in *RFC1* repeat expansion carriers are yet to be performed, we found evidence for genuine vestibular dysfunction in *RFC1*-expansion-positive individuals by caloric hyporesponsiveness in all *RFC1* repeat expansion carriers (who underwent caloric testing). The distinct underpinnings of BV in *FGF14*-linked disease warrant further in-depth investigation, including histopathology. In addition, our study confirmed further relevant features of *FGF14*-linked different from *RFC1*-linked disease, such as no relevant occurrence of chronic cough and less frequent dysarthria compared to other forms of late-onset cerebellar ataxia [8].

Besides a relatively small sample size, the present study's limitations include a potential underestimation of *RFC1*-expansion positivity, as we did not investigate sequence variants, which were recently highlighted as an additional, albeit rare, cause of *RFC1*-linked disease [17]. Moreover, the retrospective analysis results in partly incomplete data, particularly regarding somatosensory deficits, as nerve conduction studies were not available in 20% of patients. Finally, we reported only data on the horizontal VOR but not on additional vestibulo-ocular investigations, such as the visually enhanced VOR (VVOR), which should be addressed in future prospective studies.

This work suggests that BV in CA patients with *RFC1* repeat expansions is more severe as compared to CA patients with *FGF14* repeat expansions, which may find clinical utility as the severity of BV (i.e., VOR gain) may be readily measured by the vHIT and, hence, may facilitate the identification of patients with *RFC1*-repeat-expansion positivity. Additionally, our data raise the possibility that BV in *FGF14* repeat expansion carriers may be caused by cerebellar rather than vestibular dysfunction, although we note the aforementioned variation in the effect of floccular pathology on VOR gain; hence, this hypothesis requires further investigation.

## Data Availability

The data underlying the analyses in this study are available from the corresponding author, upon reasonable request.
